# Benefits and Challenges of Interdisciplinarity in CSCL Research: A View From the Literature

**DOI:** 10.3389/fpsyg.2020.579986

**Published:** 2021-01-14

**Authors:** Cindy E. Hmelo-Silver, Heisawn Jeong

**Affiliations:** ^1^Center for Research on Learning and Technology, School of Education, Indiana University Bloomington, Bloomington, IN, United States; ^2^Department of Psychology, Hallym University, Chuncheon, South Korea

**Keywords:** computer-supported collaborative learning, interdisciplinarity, bibliometric analysis, systematic review, educational technology

## Abstract

Computer-supported collaborative learning (CSCL) has a history of being interdisciplinary from its conception. Its beginnings have included computer scientists, psychologists, cognitive scientists, and educational researchers. These collaborations have been fruitful but have also posed challenges (Suthers et al., 2013). This article builds on the authors’ extensive review of the CSCL literature to examine the nature of interdisciplinary collaboration in CSCL research as well as an interdisciplinary CSCL workshop. Using a corpus of more than 700 CSCL articles, we reported an updated analysis for the theories and methods used in CSCL research. In addition, bibliometric analyses examined journals that publish CSCL research and are cited by CSCL research. CSCL research is published in journals that are aligned with interdisciplinary research with large contributions from educational research followed by technology related fields and social sciences. The contributions from domain knowledge journals are relatively weak. These analyses revealed disciplinary influences and uptakes of CSCL research and how they might differ across CSCL research clusters. Lastly, we provide a case example of a CSCL workshop to further demonstrate the interdisciplinary nature of the field. Through these analyses we aim to characterize the benefits and challenges of interdisciplinary collaboration in CSCL research. Interdisciplinarity has helped CSCL research to adopt multiple theories and methods to understand CSCL. While cultivating diversity, we also need to be mindful that research outcomes are exchanged and appropriated actively across participating disciplines so that our understanding of CSCL rises above individual disciplines.

## Introduction

Computer-supported collaborative learning (CSCL) has a history of being interdisciplinary from its conception (Stahl et al., 2014). Its beginnings have included computer scientists, psychologists, cognitive scientists, and educational research disciplines. These collaborations have been fruitful but have also posed challenges (Suthers et al., 2013). This interdisciplinary nature of the CSCL research has been reflected in the diversity of theories and methodological frameworks used in CSCL (Jeong et al., 2014). In this article, we build on this research and attempt to examine and characterize the nature of interdisciplinarity in CSCL research. We begin by examining the historical roots of research traditions that comprise CSCL research. We will then examine the interdisciplinarity of CSCL research from multiple perspectives: (1) composition of CSCL research methods and theoretical frameworks, (2) bibliometric research clusters that emerged based on shared reference citations, (3) disciplinary associations of the journals that publishes CSCL research and is cited by CSCL research, (4) disciplinary affiliations/compositions of the contributing authors to International Journal of Computer-Supported Collaborative Learning (ijCSCL), and 5) a case example of an interdisciplinary CSCL workshop. These analyses rely on an updated corpus of CSCL literature that covers ten years of research between 2005 and 2014 and recent publications in the *ijCSCL*. We also relied on a range of analyses from content analysis, bibliometric analyses, and a qualitative case example. The case example moves from a bird’s eye view of the field to a ground level description of how interdisciplinary collaboration results in new insights for the field. To set the context for the research questions and analyses that follow, we begin with a historical overview of the interdisciplinary beginnings of CSCL.

## Historical Overview

The origin of the CSCL field dates back to the 1980s. Part of what makes this fundamentally interdisciplinary as a field is the relationship between the technology in the form of computational objects and the social interactions involved in learning (Stahl et al., 2014; Ludvigsen et al., in press). CSCL is the result of several converging forces. First, it was propelled by the research in developmental and social psychology and educational research that demonstrated that students working in pairs often performed better than those who worked alone (O’Donnell and O’Kelly, 1994; Miyake, 2007). These findings propelled researchers to examine underlying mechanisms of collaboration. Educators were also keen to develop instructional arrangements to promote the effects of collaborative work (Cohen, 1994; O’Donnell and King, 1999).

Another force that has contributed to the development of CSCL is the development of technology. It connected learners across geographical regions, enabling them to interact with learners and experts who are outside the geographical and temporal range of their social interactions. This interaction was mediated by a number of computational artifacts. A number of technologies and tools had been developed to help learners engage in collaborative sensemaking activities (Miyake, 2007). Other technologies provided opportunities for rich contexts that support collaboration (Roschelle, 1992; Goldman-Segall and Maxwell, 2002; Hmelo-Silver et al., 2016). Lastly, the integration of socio-cultural theory was critical. It provided a framework to incorporate both collaboration and tool mediation.

These forces created tensions and conflicts from the beginning. Some found the significance of CSCL in its epistemological underpinnings and argued that it initiated a new paradigm of learning research (Koschmann, 1996). In contrast, others took a more pragmatic approach and saw CSCL as a way to promote learning without necessarily signing up for its radical epistemological underpinnings. Educators, for example, saw that as an instructional intervention to promote cognitive learning outcomes both within and outside of the classrooms. Technology has provided ways to make classroom collaboration more engaging and meaningful. And yet to realize its promise, CSCL needs to build on both advanced technological innovation and deep understanding of how people learn. These multiple motivations and visions for CSCL have made CSCL research interdisciplinary and prompted CSCL to adopt a diversity of theoretical and methodological approaches (Jeong et al., 2014).

In this article we address two research questions:

(1) To what extent is CSCL an interdisciplinary research community?
(2) How can different sources of evidence be used to paint a picture of interdisciplinary collaboration?

## Methods

### Article Selection and Screening

Much of the literature discussed is a secondary analysis of systematic reviews presented in earlier publications (Jeong et al., 2014, 2019a; McKeown et al., 2017) and builds on a corpus of CSCL literature collected for that purpose, the dates of that review being from 2005 to 2014. The corpora used for the systematic reviews of CSCL literature were constructed based on two databases, ERIC and Web of Science, in addition to seven key journals regarded by experts (Jeong et al., 2014) to be leaders in publishing CSCL research: *Computers and Education*, *Computers in Human Behavior*, *International Journal of Computer Supported Collaborative Learning*, I*nternational Journal of Artificial Intelligence in Education*, *Journal of Computer Assisted Learning*, *Journal of Learning Sciences*, and *Learning and Instruction*. We screened over 1,600 articles published between 2005 and 2014 to ensure each article met the following criteria: (a) STEM education, (b) empirical research, and (c) use of technology to support collaborative learning. CSCL research refers to research articles in which participants learned collaboratively with the support of computers and/or other technologies. The technology also needed to be specific so that studies examining technology integration or adoption in general were not included. Studies about students with physical or learning disabilities were excluded because these can involve special technologies not typical in CSCL. Learners needed to interact in small groups or in some ways with peers at some point during the learning process. Studies needed to address learning, broadly defined (see Jeong et al., 2014 for additional details). We defined empirical research to refer to studies that relied on first-hand data to validate a theory, hypothesis, research question, and/or design. Although we broadly used the same criteria to screen and select articles for the corpus, changes in the research question over the years had led to the construction of a corpus with a slightly different scope and nature. In Jeong et al. (2014), we covered CSCL articles from 2005 to 2009 and included CSCL research both in STEM and non-STEM (*n* = 400). Funded by the United States National Science Foundation, McKeown et al. (2017) aimed to examine CSCL in STEM domains and thus focused on only STEM CSCL research, but expanded the corpus up to 2014 to include ten years of research (*n* = 735). Jeong et al. (2019b) combined the two corpora for bibliometric analysis (*n* = 869), which is basically the articles in the corpus used by McKeown et al. (2017) with an addition of non-STEM articles in the earlier corpus by Jeong et al. (2014) that were excluded in the McKeown et al. (2017) corpus. It was done to ensure a large enough corpus for the bibliometric analysis, but made the corpus used for bibliometric analyses unbalanced because non-STEM articles were not present for the 2010–2014 period. These features of the corpora need to be taken into consideration when interpreting the results.

For the *International Journal of Computer-Supported Collaborative Learning* journal analysis, we selected multiple-authored ijCSCL articles from three time points which are 2006–2007, 2012–2013, and 2018–2019. These three 2-year periods were selected from the total number of complete issues of the journal which began its publication in 2006. This allowed examining historical trends over time and provided a view of the early issues and most recent with the 2012–2013 providing a midpoint.

### Coding

The articles that met our initial criteria were coded based on several dimensions. In our earlier examination of CSCL research practices, we examined CSCL research methods in terms of research design, research settings, data sources, and analysis methods (Jeong et al., 2014). We additionally examined how these methodological practices related to theoretical frameworks of the research. Theoretical frameworks referred to perspectives that guided the research (Danish and Gresalfi, 2018). The initial list of frameworks was derived from keywords used for the CSCL 95 conference and then expanded based on the frameworks that were represented in thearticles. Information processing theory referred to traditional cognitive theories with a strong emphasis on individual cognitive processes. Socio-cognitive theory referred to theories related to constructs of cognitive conflict and conceptual change (DeLisi and Golbeck, 1999). Constructivism referred to a broad range of theoretical approaches that emphasize active learner processing and knowledge construction in individualistic and collaborative settings (von Glasersfeld, 1995; Chi and Wylie, 2014). Socio-cultural theory referred to a diverse range of theories such as Vygotskian approaches, distributed cognition, or activity theory that emphasizes the fundamental role of tools, activities, social norms and systems (Danish and Gresalfi, 2018). Communication theory referred to theories addressing linguistic and communicative aspects of collaboration (Krauss et al., 1990). Social psychology theory referred to theories that focused on social aspects of collaboration such as status difference, gender, and/or group dynamics (Levine and Thompson, 1996). Motivation theory referred to theories with a focus on motivational aspects of learning, addressing issues such as attribution or self-regulation (Pintrich, 1999). The Other theory category referred to theories that did not fit into any of the categories that we have described (e.g., constructionism). Studies coded as Atheoretical referred to investigations that were primarily guided by practical concerns (e.g., program evaluations). Boundaries of different theoretical frameworks were not always clear-cut. If authors explicitly named their theoretical frameworks, we coded them as such. If they were not, we relied on references and major variables examined in the study (e.g., conceptual change is a typical variable or topic of study strongly associated constructivism). Studies could have more than one theoretical framework. Methodological practices refer to research design approaches (e.g., experimental, descriptive, and design-based research) and analysis methods (e.g., qualitative, quantitative, mixed methods; see Jeong et al., 2014; Hmelo-Silver and Jeong, in press, for further details). Two raters independently coded 20% of the sample with an overall kappa of.68, showing substantial agreement.

For the case study of the workshop, the first author went through the list of participants and the workshop report, identifying the academic disciplines of each of the participants based on their academic departments. Additional information regarding the workshop is drawn from the workshop report (Hmelo-Silver, 2019).

## Theories and Methods Used in CSCL

The different methods and the need to incorporate them is part of what makes CSCL a multidisciplinary field (Stahl et al., 2014). Analysis of methodological traditions in the field demonstrate that this has been the case from the beginning (Jeong et al., 2014). We coded these features of research for five years of CSCL research from 2005 to 2009 and found that the overall CSCL research practices are quite diverse, likely to reflect the diverse traditions that contributed to the formation of CSCL. We also found that this led to the use of research methods that are quite eclectic. For example, experimental work in classroom or online settings and wide usage of mixed studies. These trends were observed widely regardless of research traditions, but there was a clear alignment between research methods and theoretical frameworks. According to Jeong et al. (2014), four clusters of research emerged. Two of them were clearly guided by theoretical frameworks such as sociocultural and constructivists perspectives. While these traditions mostly relied on descriptive designs in classroom settings, there is a small cluster of CSCL research that strongly relies on experimental approaches. An updated analysis described in Hmelo-Silver and Jeong, in press) shows that the trends to use diverse methodological framework continued throughout the expanded time period (2005–2014), with a mix of methods drawn from psychology, linguistics, anthropology, and human–computer interaction.

Table 1, an updated table of theoretical frameworks that includes publications through 2014 (in STEM domains) indicates that articles with multiple theoretical frameworks account for 30% of the articles in the corpus. The largest overlap was among articles coded as constructivism and those coded as sociocultural (*n* = 26). Thus, the field continues to use diverse methodological frameworks in CSCL but since the earlier analysis, more individual articles use multiple frameworks.

**TABLE 1:** Co-occurrence of theoretical frameworks.

	Single	Multiple
Information processing	33	17
Socio-cognitive	22	22
Constructivism	188	71
Sociocultural	109	54
Communication	19	19
Social Psych	44	24
Motivation	26	18
Other	53	24
Atheoretical	77	1
Total	571	250
	69.55%	30.45%

The presence of diverse theoretical and methodological frameworks confirms that different disciplines contribute to CSCL research. The co-existence of these frameworks is reflective of the diverse research traditions that converge on CSCL and the interdisciplinarity of CSCL, but it is only a small piece of the picture. Another way to take advantage of this corpus to examine the (interdisciplinary) nature of CSCL as a field is through an analysis of the bibliometric data.

## Nature of CSCL Interdisciplinarity: Bibliometric Analysis

Bibliometrics or scientometrics in particular analyzes scientific publications to measure and understand scientific research practices. It relies on citation or other statistical data related to academic publications. The development of digital technology and large databases such as Web of Science (WOS) and Scopus has contributed to its recent rise. It is increasingly used to understand questions such as the impact of specific research fields, a set of researchers or particular publications that connects different research fields, and/or publications with large impacts (Mingers and Leydesdorff, 2015). Bibliographic coupling (BC) analysis is a kind of bibliometric analysis that analyzes references of publications and identifies clusters of articles with shared references. This technique has been used to successfully map the networks of researchers in scientific institutions or in a given research field (Grauwin and Jensen, 2011; Grauwin et al., 2012).

**FIGURE 1:**
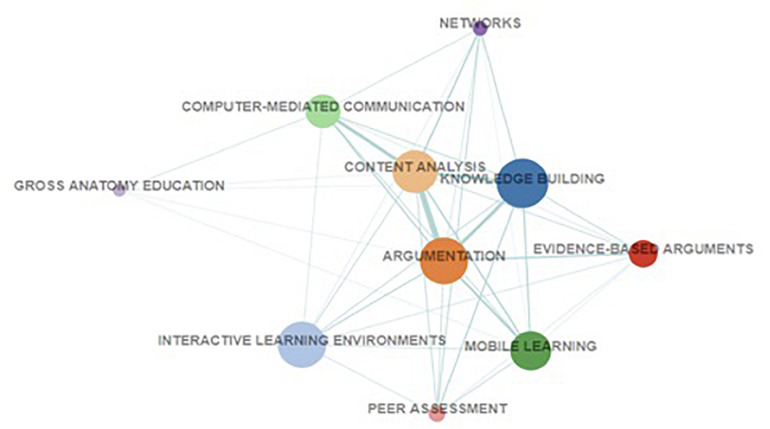
Ten CSCL BC clusters (cluster labels represent most frequently used keywords by the articles in the clusters).

Jeong et al. (2019b) have applied BC analysis to understand how CSCL research publications are organized. Using the extended corpus of CSCL articles (*n* = 869), they identified clusters of CSCL research that are linked by shared references. This is the expanded corpus mentioned in the previous section that included non-STEM articles to capture CSCL research clusters more widely. The BC analysis creates links between articles when they shared references (Kessler, 1963). A community detection algorithm based on modularity optimization (an implementation of the Louvain algorithm) was then applied to partition networks of linked articles into clusters in a map, in which a node represents a cluster with its size proportional to the quantity of articles within the clusters. Note that not all articles shared references with other articles. Clusters were not always connected to the rest of the clusters. In the end, 735 articles were included in the BC map shown in Figure 1. The rest of them (*n* = 134) did not share references with other CSCL articles, suggesting that there is some research that we have classified as CSCL that does not build on this literature and may draw on other research foundations.

The cluster labels were derived automatically based on the most frequently used keywords. Each cluster represents sub-areas of CSCL research that are linked by distinct sets of shared references, suggesting that they are referencing different knowledge bases in CSCL research. Keywords frequently used by the articles within the clusters are used as cluster labels. The “knowledge building” cluster in Figure 1 means that articles in the clusters are likely related to research relevant to knowledge building in some way. The clusters also differ in size, suggesting that some topics have been the subject of more published research than others. As Figure 1 shows, more research has been published on knowledge building (*n* = 145) and argumentation (*n* = 127) than topics such as peer assessment (*n* = 13) and gross anatomy education (*n* = 7). Jeong et al. (2019b) identified the five biggest clusters as major and the rest as minor CSCL research clusters. The five major clusters represent major areas of CSCL research such as knowledge building and argumentation, whereas the five minor clusters represent less well represented areas of CSCL research such as peer assessment and gross anatomy education. They differed in the references they share as well as in the publications sources in which their references were published.

References can reveal the intellectual traditions and disciplinary knowledge base that CSCL research draws upon. Articles in the same disciplines or sub-areas tend to cite similar publications. Another important marker of disciplinary association is the journals in which the article is published. Journals are outlets of academic research conducted in a particular field of research. They serve as gatekeepers of research and decide whether a particular piece of research is appropriate to their mission in terms of topics as well as quality (Crane, 1967). In this section, we examined the sources of research that CSCL cites and outlets of research that CSCL publishes to understand the disciplinary influences and composition of CSCL research and whether and/or how this reliance on particular disciplines may differ across sub areas of CSCL research. The historical disciplinary influences might still be visible to some degree as we have witnessed in the different theoretical approaches and methods.

To examine these questions, we extracted the following information from the articles (*n* = 735) included in the ten CSCL clusters: (1) authors, (2) year of publication, and (3) publication source (i.e., journals in which the article is published) (4) reference sources in the reference list (e.g., books, journals, and other sources that the article cites), (5) discipline categories assigned by WOS called “WOS categories.” We extracted the meta-data of the indexed articles from WOS, but hand coded the meta-data using the pdf files for the articles that were not indexed in WOS.

### Journals That Publish CSCL

Computer-supported collaborative learning journals refer to those journals that are the sources of CSCL research articles reviewed here. We identified 33 such journals based on the most frequent publication sources across the ten CSCL research clusters. Table 2 lists the top ten journals that published CSCL research during this period. They are the major outlets for CSCL research. Different journals publish different numbers of issues and articles each year. Journals with higher numbers and percentages are likely to be those journals that publish more issues and articles over the years.

**TABLE 2:** List of top ten journals publishing CSCL research.

	Journals	Aims and Scope	*N* (%)
1	Computers and Education (C&E)	“Pedagogical uses of digital technology, where the focus is broad enough to be of interest to a wider education community”	333 (38%)
2	Computers in Human Behavior (CHB)	“dedicated to examining the use of computers from a psychological perspective”	133 (15%)
3	Journal of Computer-Assisted Learning (jCAL)	“…covers the whole range of uses of information and communication technology to support learning and knowledge exchange.”	90 (10%)
4	International Journal of Computer-Supported Collaborative Learning (ijCSCL)	“A forum for a diverse range of disciplines such as education, computer science, information technology, psychology, communications, linguistics, anthropology, sociology, and business”	87 (10%)
5	Journal of the Learning Sciences (JLS)	“A multidisciplinary forum for research in education and learning…”	28 (3%)
6	Learning and Instruction (L&I)	“As an international, multi-disciplinary, peer-refereed journal,…a platform for the publication of the most advanced scientific research in the areas of learning, development, instruction and teaching”	25 (3%)
7	International Journal of Artificial Intelligence in Education (ijAIED)	“…publishes articles concerned with the application of AI to education”	24 (3%)
8	Educational Technology and Society (ET&S)	“…publishes the research that well bridges the pedagogy and practice in advanced technology for evidence-based and meaningfully educational application”	10 (1%)
9	Journal of Science Education and Technology (JSET)	“An interdisciplinary forum for the publication…that address the intersection of science education and technology with implications for improving and enhancing science education at all levels across the world”	8 (1%)
10	Anatomical Sciences Education (ASE)	“An international forum for evidence-based exchange of ideas, opinions, innovations, and research on topics related to education in the anatomical sciences of gross anatomy, embryology, histology, neurosciences, biomedical, and life sciences.”	8 (1%)

*Percentage refers to base of total number of articles in the expanded corpus (n = 869).*

We first examined the “aims and scope” statement of these journals as listed on the journal homepage to understand the disciplinary or interdisciplinary associations of the journals as identified by the editorial teams of the journals. These statements anchor the positions and directions of the journals and can serve as important guidelines for both authors and readers of the journals. We did not engage in formal coding, but looked for words or phrases that signaled associations with specific disciplines or interdisciplinary research. Most of the journals emphasize problems or research topics (e.g., “application of AI to education”) rather than disciplinary associations. *Computers and Education*, for example, states that it welcomes research articles on the “pedagogical uses of digital technology, where the focus is broad enough to be of interest to a wider education community.” Such emphasis on research problems and topics are indicative of the openness to approaches coming from different disciplines. Some journals go a step further and are explicit about this. *ijCSCL*, for example, states that it “aims to serve as a forum for a diverse range of disciplines such as education, computer science, information technology, psychology, communications, linguistics, anthropology, sociology, and business.” Not all journals have multidisciplinary orientations, however. *Computers in Human Behavior* (CHB), for example, was clear that this journal is “dedicated to examining the use of computers from a psychological perspective.” This disciplinary focus was more likely to be the case in journals focused on science and anatomy education. Still, such explicit mono-disciplinary association is an exception rather than a rule. In sum, it appears that most CSCL articles are published in journals that explicitly promote multidisciplinary approaches or emphasize research problems rather than specific disciplinary approaches.

Every journal or book indexed in WOS is assigned to at least one subject area category such as education or psychology. There are 256 WOS subject categories as of 2018^1^. WOS categories are quite detailed. There are three WOS categories for education, for example: “Education, Educational Research,” “Education, Scientific Disciplines” and “Education, Special”. Psychology has 11 WOS categories such as psychology, experimental, social, and so on. In order to examine the disciplines at a broad level more used in everyday discussion of disciplines, we grouped the WOS category of CSCL journals into four discipline groups: (1) Education (2) Technology (3) Social Sciences and Psychology, and (4) Knowledge Domains. Table 3 shows how our discipline groups map onto the WOS categories with some example journals in each group. Two journals were not indexed in WOS and thus could not be assigned to a discipline group in Table 3, but the rest of the 31 journals were assigned to at least one discipline group.

**TABLE 3:** Web of science (WOS) categories of journals.

Discipline groups	WOS subject category of Major CSCL journals	Example Journals
Education	Education and Educational Research Education, Scientific Disciplines	JCAL C&E*, ijCSCL*, JLS* IEEE Transactions on Education*
Technology	Computer Science, Interdisciplinary Applications Information Science and Library Science Computer Science, Hardware and Architecture Computer Science, Information Systems Computer Science, Software Engineering Computer Science, Theory and Methods	C&E*, ijCSCL* Computer Applications in Engineering Education* IEEE Transactions on Education*
Social Sciences and Psychology	Businesses Communication Management Psychology, Experimental Psychology, Multidisciplinary Psychology, Educational Sociology	American J of Sociology CHB Communication Research Organization Science JLS*, Learning and Instruction*
Knowledge Domains	Anatomy and Morphology Biology Engineering, Electrical and Electronic Engineering, Multidisciplinary Ergonomics Health Care Sciences and Services Medicine, General and Internal Physiology	Annals of Anatomy Behavior Information Technology Croatian Medical Journal American Biology Teacher* Computer Applications in Engineering Education*

“*” Indicates journals listed in more than one disciplinary group.

One way to define the multidisciplinarity of a journal is to examine whether they belong to more than one discipline group. CSCL publishing journals often belong to more than one discipline group. For example, journals such as *Computers and Education* and ijCSCL both belong to the Education as well as the Technology discipline groups. Computer Applications in Engineering Education (CAEE) belongs to three discipline groups: Education, Technology, and Knowledge Domains (i.e., Engineering, Multidisciplinary). About one-third (12 out of 31) of the CSCL publications are multidisciplinary in this sense. They all belong to the Education group, but varied in their second disciplinary association.

The number (and percentage) of articles in each discipline group is presented in Table 4. These numbers should be interpreted cautiously as journals often belong to more than one discipline. Even so, Table 4 shows that CSCL research is published most in journals associated with Education (81%), followed by Technology (55%), and Social Sciences (25%). Journals in the Knowledge Domains group do publish CSCL research, but only 2% of CSCL articles have been published in such journals. Considering that a quite sizable portion of CSCL research involves STEM education (Jeong et al., 2019a), this mismatch is puzzling. In spite of STEM domains dominating CSCL, CSCL may not be widely adopted as a useful pedagogical strategy and/or there might not be sufficient audiences for CSCL research in these journals. In addition, although a large number of articles are being published in technology domain journals, they are concentrated on three journals: *Computers and Education* (*n* = 333), *ijCSCL* (*n* = 87), and *Computer Applications in Engineering Education* (*n* = 1). Technology disciplines are part of CSCL research, and yet again, CSCL research is not being published as widely in technology journals. This, however, may be an artifact of the corpus, since one of the criteria to be included in the corpus was that the articles need to be empirical articles (Jeong et al., 2014). Articles that focus purely on technical or design aspects of CSCL tools were not likely to be included in the corpus. A similar pattern can be observed in Social Sciences domain journals. Most of the articles were published in five journals, most of which publish psychology or educational psychology research as Computers in Human Behavior (*n* = 133) and JLS (*n* = 28). In sum, while CSCL research is multidisciplinary in its historical origin and participating members’ disciplines, CSCL research may not be relevant to participating disciplines to the same extent. The main audiences for CSCL research are readers of education journals or journals that are at the intersection of education, technology, and neighboring disciplines.

**TABLE 4:** Discipline groups of the journals that publish CSCL research.

Discipline groups	*N* of journals	*N* (%) of articles
Education	24	568 (81%)
Technology	3	382 (55%)
Psychology and Social Sciences	5	176 (25%)
Knowledge Domains	10	14 (2%)

**FIGURE 2:**
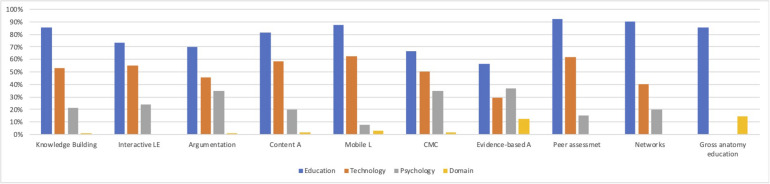
Disciplinary composition of the publishing journals by clusters.

The disciplinary composition of CSCL publishing journals may vary depending on the nature of the research question. When the use of the tools in the classroom and appropriate pedagogical interventions are the focus, it is more likely to be relevant to educational researchers and journals that publish such research. Figure 2 presents the proportion of articles published in each discipline group across the ten CSCL research clusters. Clusters are ordered from the biggest on the left to the smallest in size on the right in the figure. The proportion of each discipline group fluctuates across the clusters, but educational journals play the biggest role in publishing CSCL research, followed by technology journals and then by psychology and disciplinary education journals, replicating the general trend that we observed in Table 4. A few deviations from this general trend are notable, however. Knowledge domain journals have a larger presence in clusters such as gross-anatomy education (14.29%) and evidence-based arguments (12.20%) clusters compared with the other clusters. The cluster with the highest proportion of Knowledge Domains journals is the gross anatomy education cluster which is the smallest in size along with a narrow research focus. The cluster with the second highest proportion of knowledge domain journal articles is the evidence-based arguments cluster (12.2%) which is also relatively small in size and indicates a narrower research focus, a specific sub-type of argumentation. Taken together, it appears that the disciplinary composition of CSCL publishing journals is more or less the same across the ten CSCL research clusters, although a few deviate from it mainly due to the size and research topics of the clusters.

### Journals That CSCL Research Articles Cite

The interdisciplinarity of CSCL research can also be examined based on the journals that it cites. There are 1,885 distinct reference sources cited by the CSCL research in the corpus, which include books and book chapters, but journal articles turn out to be major citing sources of CSCL research. Table 5 below lists the top ten journals that articles in CSCL research cite. As shown in Table 5, *Computers and Education* is cited by about half of the CSCL articles; *Journal of the Learning Sciences* (JLS) and *Journal of Computer Assisted Learning* (JCAL) are cited by about one-third of the CSCL articles. *Computers and Education* (C&E) continue to be the top referenced journals as well as publication outlet.

**TABLE 5:** List of major journals that CSCL research cites.

	Journals	Frequencies (%)
1	Computers and Education (C&E)	468 (54%)
2	Journal of the Learning Sciences (JLS)	290 (33%)
3	Journal of Computer-Assisted Learning	280 (32%)
4	Computers in Human Behavior	265 (30%)
5	Learning and Instruction	233 (27%)
6	ETR&D-Educational Technology Research and Development*	218 (25%)
7	Instructional Science*	208 (24%)
8	Review of Educational Research*	205 (24%)
9	British Journal of Educational Technology*	193 (22%)
10	Journal of Educational Computing Research*	175 (20%)

“*” Indicates journals that were not in Table 2. Frequencies and percentages refers to the percentages of the articles in the expanded corpus (n = 869).

Comparison between Tables 2 and 5 shows that a group of journals such as C&E and JLS appear in both tables, indicating that they play an important role both as an outlet and reference source of CSCL research. At the same time, a group of journals emerged as a major reference source of CSCL research in Table 5 although they did not appear in Table 2. ETR&D, Instructional Science, and Review of Educational Research fall into this category. In the case of Review of Educational Research, it publishes only review articles and thus is not likely to be a publication outlet for primary empirical articles included in our corpus. ETR&D and Instructional Science do publish CSCL research (ranked 9th and 19th in the publishing journal list), but did not appear in Table 2 likely due to their low volume of publications. Yet there is another group of journals that appear in Table 2, but not in Table 5. For example, ijCSCL appears in Table 2, but not in Table 5. This is likely because it began publishing in 2006 and there is likely to be a time lag until researchers start reading and referencing articles from. ijAIED and ET&S are also journals that publish CSCL articles, but they are not referenced frequently in CSCL research. It may indicate uneven readership interests so that there is likely to be more interest in the application of technology to support CSCL in the AIED community, although computer science and technology articles may not be actively cited and referenced in the rest of the CSCL research.

Nonetheless, the disciplinary composition of the citing journals largely remains more or less the same as the disciplinary composition of the publishing journals (see Table 6). We analyzed the disciplines of the 39 citing journals included in the CSCL BC Map, excluding books or non WOS journals and journals cited by little CSCL research. Most of its citations are from education journals, indicating that CSCL research substantially builds on educational research. This does not mean that research from other disciplines does not contribute to CSCL research. A sizable proportion of the citation comes from journals in technology and/or social sciences journals as well as from journals in the knowledge domains, even though it is a proportionally small part of CSCL citations. The diverse historical origins of CSCL is visible from the disciplines of the citing journals, but knowledge uptake across disciplines appears to be uneven.

**TABLE 6:** Discipline groups of the journals that CSCL research cites.

Discipline groups	*N* of journals	*N* (%) of citations
Education	25	2,019 (61%)
Technology	5	482 (15%)
Social Sciences	11	759 (23%)
Knowledge Domains	7	42 (1%)

**FIGURE 3:**
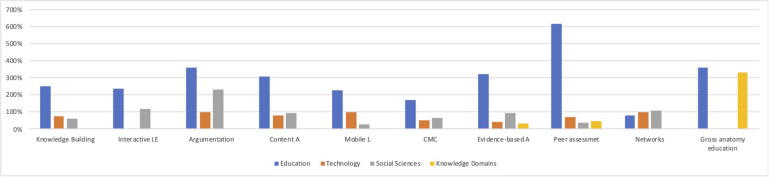
Disciplinary composition of citing journals by clusters.

We further examined the pattern of research uptake across CSCL research clusters. Figure 3 presents the percentage of citations that journals in each discipline group received across the ten CSCL research clusters. Clusters vary in terms of disciplines of the journals the research they cite belong to. Most clusters draw on research from at least three discipline groups. Peer assessment and evidence-based clusters draws on all four discipline groups. Interactive Learning Environment and gross-anatomy education draws from two discipline groups. All clusters heavily cite research in education journals, but the extent of reliance varies. In the networks cluster, it relies on technology and social science journals more and in the gross anatomy cluster domain journals were equally cited. Taken together, educational research is the major knowledge base in all CSCL research clusters, but exact disciplinary composition varies somewhat depending on the clusters.

## How Interdisciplinary Is *ijCSCL*?

Another marker of CSCL as an interdisciplinary field is through the composition of the journal devoted specifically to CSCL research. This includes the editorial team as well as the authors of articles in the journal. Academic societies often have flagship journals which members consider to be representative of the research that they do in the community. In the case of CSCL, it is the *International Journal of Computer-Supported Collaborative Learning* (*iJCSCL*). It was established in 2006 as the field was being established. There are several major journals that publish substantial amounts of CSCL research, but it has quickly established itself as a major outlet of CSCL research. This is remarkable when we consider that ijCSCL publishes far fewer articles per year than those journals. In this section, we examine the interdisciplinarity of the journal in terms of the disciplinary associations of its editorial team and contributing authors.

One example of the interdisciplinary nature of the journal is the editorial team of the *ijCSCL*. The founding co-editors included a computer scientist (Dr. Gerry Stahl) and a psychologist (Dr. Friedrich Hesse) with a similar composition among the newest co-editor team (Dr. Sanna Järvela, psychologist and Dr. Carolyn Rosé, computer scientist).

The interdisciplinary characteristics of ijCSCL can be inferred by looking at the range of the contributing authors’ disciplinary associations in multi-authored ijCSCL articles. The number and percentages of the multi-disciplinary multi-authored articles are presented in Table 7. As this table shows, the percentage of articles (excluding editorials) has ranged from 14.29 to 29.42%, and including editorials has been roughly a third of the total multi-authored contributions. These interdisciplinary teams tend to be among social scientists (psychology and education), technology (computer and information sciences), and domain-specific (e.g., STEM departments and health sciences). These numbers are promising, but there is also a long way to go to promote more interdisciplinary collaboration that supports innovation in technology and sophisticated analysis of how the technology is a tool for CSCL supporting learning and engagement.

**TABLE 7:** Interdisciplinary composition of *ijCSCL* multi-authored articles.

Year	Total # Articles including editorials	# Interdisciplinary	%	# excluding editorials	%
2006	24	7	29.17	3	14.29
2007	22	8	36.36	6	31.58
2012	27	6	22.22	5	21.74
2013	22	8	36.36	5	27.78
2018	22	7	31.82	4	22.22
2019	21	7	33.33	5	29.42

## Case Example: Building Interdisciplinary Capacity Workshop

Although the journal citations and authorships provide some evidence of interdisciplinary collaboration, they may also underestimate the coherence in the community. Many workshops that try to solve CSCL problems are broadly interdisciplinary, a recognition that to build capacity in CSCL, a combination of technological, pedagogical and methodological approaches is needed. An example of this is the workshop organized by the first author (Hmelo-Silver, 2019). The workshop had an explicit goal of “Building Interdisciplinary Capacity for Understanding and Supporting Computer Supported Collaborative Learning.” Although the particular team fluctuated over a series of four 1–2 day workshops, the regular contributors included 17 scholars who identified as education researchers and 13 who largely identified as computer scientists with some representation among industrial engineering and management sciences. This interdisciplinary group discussed actionable indicators in work on learning analytics and adaptive support for collaborative learning. Learning analytics work showed promise for informing collaborative learning but many of the indicators being used were shallow measures of participation and engagement. The next logical step for the workshops was to delve deeper and extract actionable indicators from research on collaboration, to determine whether or not supported by technology, they could be used to develop new technologies that would build models of collaboration that would be amenable to learning analytics, and ultimately lead to better adaptive support for collaborative learning, whether in stand-alone systems or to help teachers on a just-in-time basis. Many insights developed as behaviorally oriented researchers worked in small groups with more technically oriented researchers to identify what both saw as needed and interesting for driving research on CSCL forward. One outcome of this project was developing a common, shared language for talking about collaborative learning that can facilitate reporting and comparing research on collaborative learning as well as advancing joint research (e.g., Mott et al., 2019; Saleh et al., 2019; Chen et al., 2020). The workshop integrated across disciplines to show how CSCL researchers conceive of high-quality collaboration and indicators of lesser quality. This begins to provide a shared language to talk about aspects of collaboration that would be targets for automated analysis of collaboration, learning analytics, and adaptive support for collaborative learning. These discussions have led to further interdisciplinary collaboration towards just this end among learning sciences, instructional systems technology, and computer science in a team that is developing adaptive support for game-based learning (e.g., Mott et al., 2019; Saleh et al., 2020).

## Discussion

Educational research in general, and the learning sciences in particular is a multidisciplinary field that exists at the nexus of psychology, sociology, linguistics, anthropology, computer science, and technology (Lund et al., 2020; Pea and Linn, 2020). As an important branch of the learning sciences CSCL should be multidisciplinary as it needs to address educational, social, and psychological aspects of learning as well as technology designs and learning domains (e.g., disciplinary knowledge, skills, and practices) to be successful. This is reflected in the journals in which CSCL research is published and ways in which there are opportunities for interaction across disciplines. We have examined this interdisciplinarity through systematic review of the literature, bibliometric analyses, examination of editorial and authorship patterns in a major CSCL journal, and a case example from an interdisciplinary workshop. Together, these provide suggestions for ways that the CSCL research community works across disciplines, addresses interdisciplinary audiences, and where there is more that could be done.

Although CSCL began as a multidisciplinary endeavor with its research methodology and theoretical frameworks reflecting diverse traditions, the bibliometric analysis suggests that the main outlet and audience for CSCL research appears to be largely educational research journals, with some exceptions. In some sense, it is understandable. As a pedagogical strategy, there is a clear relevance to education. Still, the scarcity of journals that publish CSCL research devoted to disciplines other than education provide a barrier to CSCL research achieving its transdisciplinary goal. As the flagship journal of CSCL however, *ijCSCL* is a notable exception. Since its inception, this journal has had an international editorial team that includes computer scientists, learning scientists, educational psychologists, and discipline-based educational researchers (most notably in STEM education). This journal has generally had 20–30% of its articles composed of interdisciplinary collaborations. It is a venue that welcomes contributions from researchers across these domains (and a survey of the most recent volume of *ijCSCL* suggests at least one computer science contribution in each issue).

CSCL has been a collaboration between the technical and more socially oriented research fields. This research has appeared infrequently in journals that are dedicated to the teaching of specific disciplines such as STEM. These discipline-specific journals may be distributed across a range of fields and dilute the impact across any one field. We reported in our meta-analysis of CSCL that its effectiveness may vary depending on the learning domains and suggested that CSCL needs to be tailored to meet the needs of the knowledge domain (Jeong et al., 2019a). This may require active collaboration with disciplinary education researchers, and yet may not be well-reflected in authorship and journal outlets during the ten years of CSCL research that our corpus covers. Our analysis only covers active authorship whereas disciplinary expertise and collaboration may be reflected as contributions that are not authorship (e.g., as acknowledgments).

Nonetheless, we do see opportunities for collaboration. Many of the journals that authors publish in, the theories and research methods that draw from multiple fields, and the in-person interactions suggest that these interdisciplinary collaborations can and do occur with some regularity and are reflected in the diverse theoretical frameworks and research methods. From the early history of the field to the current journal editorship, the collaborations and contributions have been between computer scientists, educational technologists, and social scientists from the learning sciences, educational psychology, and other education fields. As a field, CSCL requires knowledge of design and pedagogy, technical expertise, classroom research strategies, and knowledge of multiple research methods. Collaborations between socially oriented researchers and technically oriented scholars can help bring more ambitious and forward thinking visions than either can alone. Computer scientists can help envision technical possibilities and advancements whereas social scientists can think about a pedagogical wish list but may not be able to envision what is technically possible.

The possibilities of these collaborations are exciting but also are challenging. Different disciplines have different standards for publication. Conference proceedings are more valued in technical areas (e.g., computer science) but less so in social sciences and education. We note that our analyses did not examine conference proceedings. In addition, the genre of research will be tied to particular disciplines (e.g., design and evaluation for computer science compared with empirical research in the learning sciences). University structures also tend to reward one publishing in one’s own disciplinary field, providing further barriers and disincentives for cross-disciplinary work. However, in our analysis, there are clearly some high-impact journals at the intersection of disciplines. Bringing people together in workshops and face-to-face conferences is one way that these interactions have been promoted (Suthers et al., 2013; Hmelo-Silver, 2019). There are serious efforts underway to highlight and promote interdisciplinary work, most notable being the International Alliance to Advance Learning in the Digital Era^2^ (IAALDE). This organization has promoted sharing research across disciplines that include behavioral, educational, and computer science fields. These organizations have committed to showcasing work across the disciplinary boundaries. This represents an interdisciplinary effort among leaders of these societies.

Computer-supported collaborative learning is a field with multidisciplinary foundations and origins (Hoadley, 2018). Through a range of analytic approaches, we have demonstrated the multidisciplinary theoretical and methodological foundations, the citations patterns, *ijCSCL* editorial and authorship collaborations, and workshop interactions to make an argument for ways in which there are influences to and from different disciplines and actual interactions among them. Although both social sciences and technical disciplines have been an important part of CSCL, the CSCL field has foundations it can build on for an even more interdisciplinary future.

## Author Contributions

CH-S worked on the sections on the systematic review, ijcscl analysis, and case example. HJ worked on the bibliometric analysis. Both authors contributed equally to the introduction and discussion.

## Conflict of Interest

The authors declare that the research was conducted in the absence of any commercial or financial relationships that could be construed as a potential conflict of interest.

## Footnotes

1 https://images.webofknowledge.com/images/help/WOS/hp_subject_category_terms_tasca.htm

2 http://www.alliancelss.com/
